# Interferon-free regimens improve health-related quality of life and fatigue in HIV/HCV-coinfected patients with advanced liver disease: A retrospective study

**DOI:** 10.1097/MD.0000000000004061

**Published:** 2016-07-08

**Authors:** Bernhard Scheiner, Philipp Schwabl, Sebastian Steiner, Theresa Bucsics, David Chromy, Maximilian C. Aichelburg, Katharina Grabmeier-Pfistershammer, Michael Trauner, Markus Peck-Radosavljevic, Thomas Reiberger, Mattias Mandorfer

**Affiliations:** a Division of Gastroenterology and Hepatology, Department of Internal Medicine III; b Vienna HIV & Liver Study Group; c Division of Immunology, Allergy and Infectious Diseases, Department of Dermatology, Medical University of Vienna, Vienna, Austria.

**Keywords:** boceprevir/PEGIFN/RBV treatment, health-related quality of life, HIV/HCV coinfection, interferon-free HCV treatment

## Abstract

Health-related quality of life (HRQoL) is impaired in HIV/HCV-coinfected patients (HIV/HCV) and further decreased by interferon (IFN)-based therapies. We aimed to investigate the impact of IFN- and ribavirin (RBV)-free therapies on HRQoL and fatigue.

Thirty-three HIV/HCV-coinfected patients who underwent HCV therapy with sofosbuvir in combination with daclatasvir or ledipasvir were retrospectively studied and compared to 17 patients who received boceprevir (BOC)/PEGIFN/RBV. HRQoL (mental [MCS] and physical [PCS] component score) and fatigue were assessed using the SF-36 (Short Form 36 Health Survey) and the FSS (Fatigue Severity Scale), respectively. HRQoL/fatigue was evaluated at baseline (BL), midway, and 12 weeks after the end of treatment (FU).

At BL, both domains of HRQoL as well as the severity of fatigue were significantly impaired in HIV/HCV, when compared to a healthy population. Already during treatment, IFN/RBV-free therapy improved physical health (PCS: 41.4 ± 9.7 vs. 47.0 ± 11.2; *P <* 0.01) and reduced fatigue (37.8 ± 14.0 vs. 31.9 ± 15.2; *P* *=* 0.01), whereas we observed a substantial worsening of both factors in patients treated with BOC/PEGIFN/RBV. Since these improvements were maintained, patients treated with IFN/RBV-free therapy reported an improvement in physical health (PCS: 41.4 ± 9.7 vs. 45.8 ± 12.7; *P* *<* 0.01) and fatigue (37.8 ± 14.0 vs. 30.9 ± 14.8; *P* *=* 0.04) at FU. While AIDS-patients had a higher severity of fatigue at BL and showed a reduction of fatigue (42.5 ± 14.0 vs. 31.6 ± 15.7; *P* *=* 0.01), mental health only improved in patients without AIDS (MCS: 35.7 ± 5.3 vs.40.7 ± 6.4; *P* *=* 0.04). HIV/HCV with severe fatigue at BL (>median BL-FSS) showed most pronounced improvements in severity of fatigue (49.7 ± 7.0 vs. 32.0 ± 16.7; *P* *<* 0.01).

In contrast to IFN-based regimens, highly effective and well-tolerated IFN-/RBV-free regimens improve HRQoL (especially physical health) and fatigue already during treatment. All patients with HIV/HCV coinfection should be considered for HCV treatment; however, patients with severe fatigue should be prioritized.

## Abstract 2

Supplemental Digital Content is available in the text

## 1 Introduction

Hepatitis C virus (HCV) coinfection is common in human immunodeficiency virus (HIV)-positive individuals resulting in an overall number of about 5 million HIV/HCV-coinfected patients worldwide.^[1]^ In contrast to HCV-monoinfected, HIV/HCV-coinfected patients are at higher risk of liver-related morbidity and mortality caused by advanced fibrosis,^[2,3]^ end-stage liver disease, and hepatocellular carcinoma.^[4]^ Thus, treatment and eradication of chronic HCV-infection represents an important clinical management priority in HIV/HCV-coinfected patients. According to current European Association for the Study of the Liver (EASL) guidelines, antiviral treatment is indicated for all HCV-positive individuals and should be prioritized in patients with HIV coinfection (due to faster fibrosis progression rates^[5,6]^) as well as in patients with high transmission risk (i.e., in case of intravenous drug abuse or among men who have sex with men).^[7,8]^ Since intravenous drug abuse and men who have sex with men represent the most important transmission routes both for HIV and HCV infections,^[9,10]^ several indications for HCV treatment are present in the majority of HIV/HCV-coinfected patients.

While HIV/HCV-coinfected patients already showed a significantly reduced health-related quality of life (HRQoL) compared to a general population,^[11]^ the previous standard of care treatment with pegylated interferon and ribavirin (PEGIFN/RBV) further compromised patients’ HRQoL^[12]^ and was contraindicated in a substantial proportion of patients due to potential severe somatic^[13,14]^ and psychiatric side effects.^[15]^ In contrast, novel IFN- and RBV-free regimens for HCV are well tolerated^[16]^ and extremely effective even in patients with HIV/HCV coinfection.^[16–18]^ Thus, allowing treatment and HCV eradication in most patients including those with decompensated disease and most pronounced impairments in HRQoL.^[19]^ However, currently there are no data on the course of HRQoL and severity of fatigue measured by standardized questionnaires in HIV/HCV-coinfected patients undergoing IFN and RBV-free treatments. Therefore, we analyzed HRQoL and severity of fatigue before, during and after IFN- and RBV-free HCV-treatment in comparison to a historical control of patients receiving the first generation direct acting antiviral (DAA) boceprevir (BOC) in combination with PEGIFN/RBV.^[20]^

## 2 Methods

### 2.1 Study design

Thirty-three HIV/HCV-coinfected patients with advanced liver disease (n = 31, 93.9%) or severe extrahepatic manifestation (n = 2, 6.1%), who were treated at the Medical University of Vienna, were retrospectively analyzed. Most patients (n = 31, 93.9%) received sofosbuvir/daclatasvir (SOF/DCV) while 2 patients (6.1%) were treated with sofosbuvir/ledipasvir (SOF/LDV). The decision to use either SOF/DCV or SOF/LDV was at the physician's discretion.

### 2.2 Evaluation of liver disease

In line with our previous study,^[21]^ advanced liver disease and cirrhosis were diagnosed by transient elastography (BL liver stiffness >9.5 and >12.5 kPa,^[22]^ respectively), liver biopsy (METAVIR >F2 and F4, respectively), or hepato-venous pressure gradient (HVPG) measurement (portal hypertension [≥6 mmHg] and clinically significant portal hypertension [≥10 mmHg], respectively). Patients presenting with clinically significant portal hypertension and/or liver stiffness 10 kPa or more, were screened for overt hepatic encephalopathy (HE) and portosystemic collaterals^[23]^ by clinical assessment and evaluation of blood ammonia levels (upper normal limit: 50 μmol/L)^[24]^ as well as radiological imaging, respectively.

### 2.3 Antiviral treatment

Patients received 400 mg SOF (Sovaldi, Gilead, Cambridge) once daily plus DCV (Daklinza, Bristol-Myers Squibb, Uxbridge, UK; n = 31, 93.9%), 2 patients received the fixed dose-combination SOF 400 mg plus LDV 90 mg (Harvoni, Gilead, Cambridge; n = 2, 6.1%) once daily according to ION-1 study.^[25]^ Once-daily dosing of DCV was performed according to the ALLY-2 study^[16]^ with 30, 60, or 90 mg/d depending on the concomitant antiretrovirals used for HIV treatment. The following treatment duration regimens were used for SOF/DCV-therapy: HCV-GT 1/4 without cirrhosis: 12 weeks, HCV-GT 1/4 with cirrhosis: 24 weeks; HCV-GT 3: 24 weeks. SOF/LDV-therapy was given 24 weeks for GT-1 treatment-experienced with cirrhosis, and 24 weeks for GT-3 treatment-naïve without cirrhosis. If HCV-RNA was still detectable 4 weeks before the planned end of treatment, treatment was extended for 4 additional weeks.^[21]^ High HCV-RNA was defined as 6∗10^6 IU/mL or more as previously described.^[26]^

### 2.4 Assessment of physical and mental health-related quality of life

HRQoL was assessed using the Short Form Health Survey (SF-36). This questionnaire is well-validated both for HCV monoinfection^[27,28]^ and HIV/HCV coinfection,^[12]^ and has shown good reproducibility and reliability.^[29]^ The SF-36 consists of 36 questions summing up to 8 domains of HRQoL: Physical Functioning (PF), Role-Physical (RP), Bodily Pain (BP), General Health (GH), Vitality (VT), Social Functioning (SF), Role-Emotional (RE), and Mental Health (MH). Physical (PCS) and Mental (MCS) Component Score—the 2 main statements of the SF-36 questionnaire—were calculated according to SF-36 user manual.^[30]^ Norm-based scaling was applied to all subscales and the 2 component scores in reference to a healthy general population with an average value of 50 points. A score between 45 and 55 points represents a physiological (“normal”) HRQoL, while a value less than 45 indicates “worse” and a value more than 55 “better” HRQoL, respectively. A clinically important change was defined as a change of ≥ 4.2 points from baseline (BL) as described by Samsa G et al.^[31]^ and Spiegel BM et al.^[32]^ Patients were asked to fill out the SF-36 questionnaire at baseline, midway, and 3 months after the end of treatment (at the clinical visit for assessment of sustained virological response 12 weeks after treatment cessation, SVR12).

### 2.5 Assessment of severity of fatigue

Fatigue severity scale (FSS) was used to evaluate fatigue—a common symptom of chronic HCV infection—at baseline, midway, and 3 months after treatment cessation. This tool is well validated^[33]^ and has frequently been used in the setting of HCV monoinfection^[34]^ and HIV/HCV coinfection.^[12]^ The lowest value (9 points) represents no fatigue, 30 points or more indicates pathologic fatigue and a value of 50 points or more shows debilitating fatigue with a maximum score of 63 points.^[35]^ We compared 2 groups with high versus low BL-fatigue classified by the median BL-FSS-value of 39.5 points.

### 2.6 Comparison of changes in HRQoL and severity of fatigue under IFN- and RBV-free therapy versus boceprevir-based triple therapy

This comparison was performed based on HRQoL (SF36) and fatigue (FSS) assessments from a historical control previously reported by Mandorfer et al.^[20]^ Seventeen HIV/HCV-coinfected patients receiving triple therapy with PEGIFN/RBV and the first-generation DAA BOC reported their HRQoL and fatigue as described earlier.

### 2.7 Statistical analysis

Statistical analyses were performed using IBM SPSS 23.0 (IBM, Armonk, NY) and GraphPad Prism (GraphPad Software, La Jolla, CA). Categorical values were reported as numbers (proportions) of patients with the certain characteristic. Continuous variables were reported as mean (±standard deviation) or median (range). Comparisons of HRQoL-/FSS values between different time points were performed using a paired *t*-test or Wilcoxon signed-rank test as applicable, whereas comparisons between different treatment groups were performed using independent *t*-test or Mann–Whitney *U* test, respectively. Correction for potential differences in baseline values was performed by ANCOVA. Categorical values were analyzed using Pearson's Chi-squared test or Fisher's Exact test. A *P* value of 0.05 or less was considered statistically significant.

### 2.8 Ethics

This study was approved by the ethics committee (EK 1814/2015) of the Medical University of Vienna and conducted following the Helsinki Declaration (version 2013^[36]^).

## 3 Results

### 3.1 Patient characteristics

Overall, 33 HIV/HCV-coinfected patients (Table 1) were included in this analysis on changes of HRQoL during and after novel IFN- and RBV-free HCV therapy. The majority of patients were male (63.6%) with a mean age of 49.9 ± 7.6 years at treatment initiation. Two-thirds (66.7%) of HIV patients had HCV GT-1, whereas 8 (24.2%) and 3 (9.1%) patients had HCV-GT 3 and HCV-GT 4 coinfections, respectively. A history of alcohol abuse was found in 7 patients (21.2% of the cohort). Most patients (n = 31, 93.9%) presented with advanced liver fibrosis, whereas 2 (6.1%) patients were treated due to severe extrahepatic HCV manifestations. All but one patient received concomitant antiretroviral therapy (ART). Immune status was well preserved with a median CD4+ T-lymphocyte (CD4+) count of 482 (75–553) cells/μL.

**Table 1:**
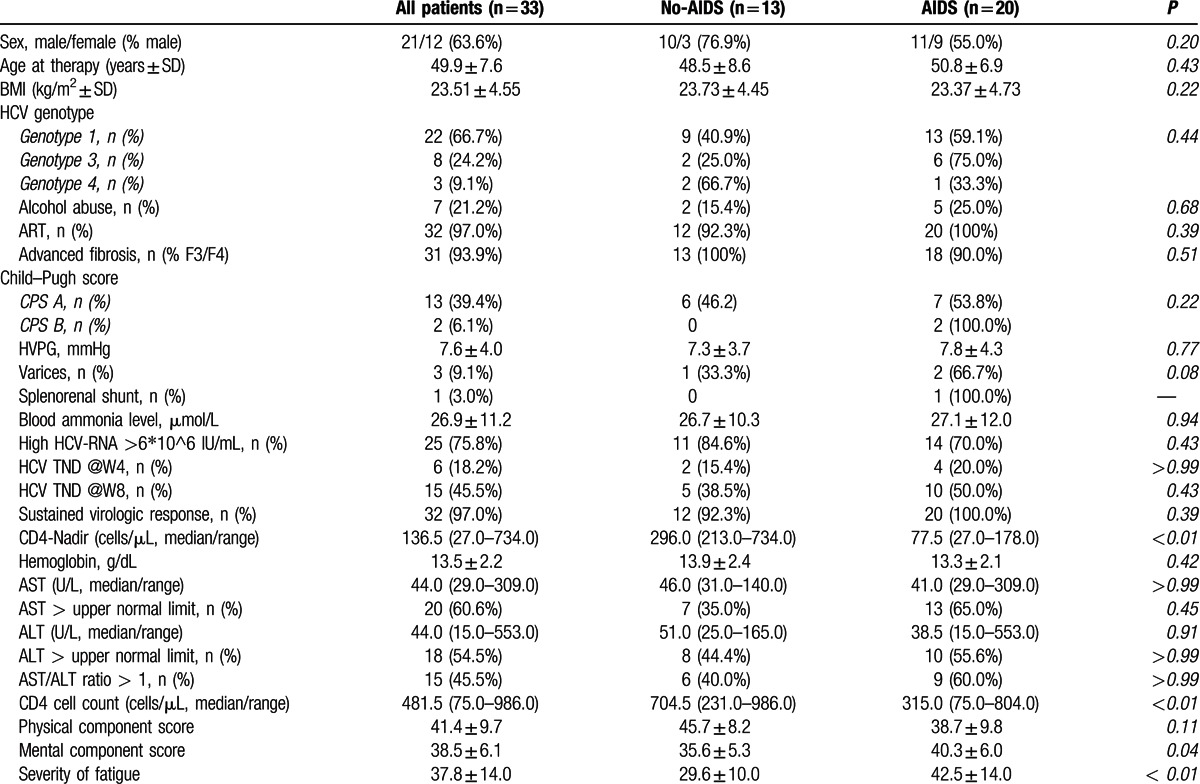
Demographics and comparison between patients with and without AIDS.

### 3.2 Severity of liver disease, portosystemic collaterals, and hepatic encephalopathy

About 45.5% (15/33) of patients had cirrhosis. Among patients with cirrhosis, 86.7% (13/15) were CP stage A, while 13.3% (2/15) had CP stage B cirrhosis. No patient with CP stage C was included.

Information on HVPG was available in 81.8% (27/33) of patients. Portosystemic collaterals (esophageal varices [n = 3] and a splenorenal shunt [n = 1]) were observed in 36.4% (4/11) of patients with or at risk for clinically significant portal hypertension (CSPH; HVPG ≥ 10 mmHg). Thus, 12% (4/33) of the overall study population had portosystemic collaterals. Only 1 patient had overt episodic HE type C^[37]^ and elevated blood ammonia levels prior to initiation of antiviral therapy. HE treatment was not changed during the study period.

### 3.3 Changes in HRQoL

**Figure 1:**
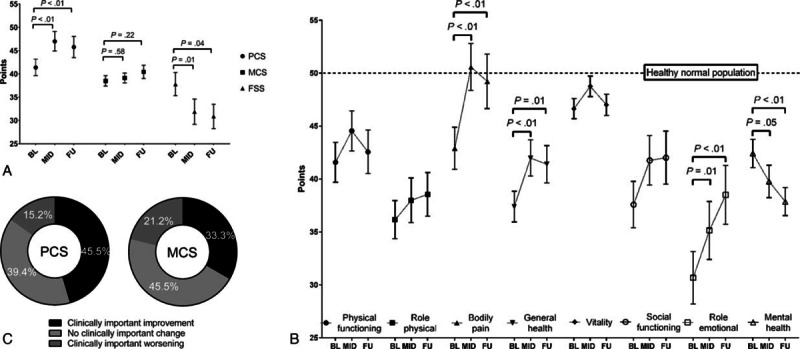
A, Physical and mental health, and severity of fatigue at baseline, mid-treatment, and follow-up. B, SF-36 subscales at baseline, mid-treatment, and follow-up. C, Proportion of patients with clinically important changes in physical and mental health from baseline to follow-up. Continuous variables shown as mean ± SEM. BL = baseline, FSS = fatigue severity scale, FU = follow-up, MCS = mental component score, MID = mid-treatment, PCS = physical component score.

At baseline (BL) the 2 global dimensions of HRQoL, physical (physical component score, PCS) and mental (mental component score, MCS) health were significantly impaired when compared to a normal healthy population (set to 50 points) with mean values of 41.4 ± 9.7 and 38.5 ± 6.1 points, respectively (Fig. 1).

Physical health (PCS) already improved statistically significantly (PCS at MID: 47.0 ± 11.2; *P* < 0.01) during IFN-/RBV-free therapy. This improvement was sustained at follow-up (PCS at FU: 45.8 ± 12.7; *P* < 0.01). In contrast, mental health (MCS) values did not change statistically significantly during the treatment period (MCS at MID: 39.1 ± 5.7; *P* = 0.58) nor after cessation of treatment (MCS at FU: 40.4 ± 7.9; *P* = 0.22).

Twenty-one (63.6%) and 19 (57.6%) patients showed a numeric improvement in PCS and MCS from BL to FU. Figure 1C summarizes the proportions of patients with clinically significant changes versus no relevant changes of physical and mental health. While almost half of the patients (45.5%) experienced a clinically important improvement of PCS, only one third of patients (33.3%) showed an improvement in mental health (MCS). Only 15.2% and 21.2% of patients reported a clinically important worsening of PCS and MCS at FU, respectively. Changes in severity of fatigue will be discussed later.

### 3.4 Changes in subcategories of HRQoL (SF-36 subscales)

The following 3 subscales improved significantly from BL to FU (Fig. 1B):

bodily pain (BP at BL: 42.9 ± 11.0; BP at FU: 49.2 ± 14.5; *P* *<* 0.01)
general health (GH at BL: 37.4 ± 8.1; GH at FU: 41.4 ± 9.9; *P* *=* 0.01)
role emotional (RE at BL: 30.7 ± 13.5; RE at FU: 38.5 ± 15.7; *P* *<* 0.01)

No statistically significant changes were observed in the other 4 subscales physical functioning (PF), role physical (RP), vitality (VT), and social functioning (SF). Interestingly, there was a significant stepwise deterioration of the mental health subscale (MH) from baseline (MH at BL: 42.4 ± 7.4) to mid-treatment (MH at MID: 39.8 ± 8.4; *P* *=* 0.05) and to follow-up (MH at FU: 37.9 ± 7.4; *P* *<* 0.01).

### 3.5 Changes in severity of fatigue

**Figure 2:**
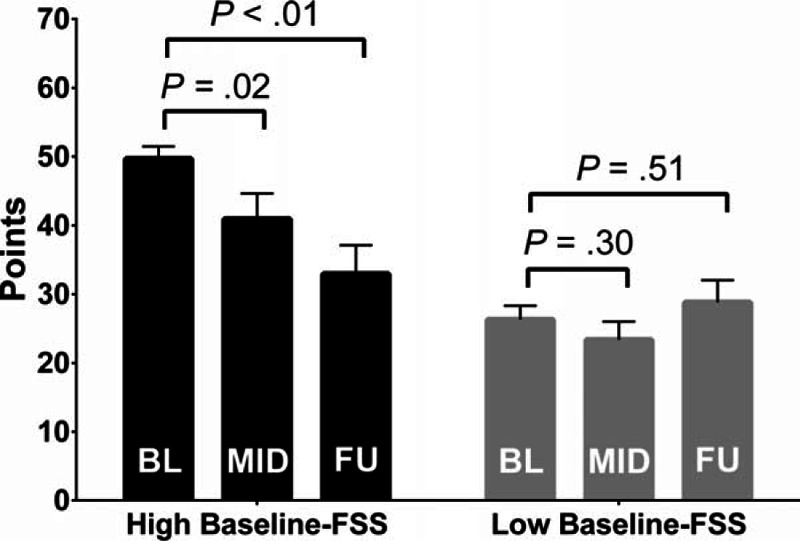
Comparison of fatigue between patients with high versus low fatigue scores at baseline (≥ median BL FSS values); continuous variables shown as mean ± SEM. FSS = fatigue severity scale.

We next analyzed severity of fatigue at baseline, midway, and follow-up (Figs. 1A and 2). At BL, the patients showed elevated mean FSS values of 37.8 ± 14.0 points, representing a pathological fatigue state. The reported severity of fatigue significantly decreased to 31.9 ± 15.2 (*P* *=* 0.01) during treatment and remained improved at follow-up (FSS at FU: 30.9 ± 14.8; *P* *=* 0.04).

When comparing patients with high versus low fatigue levels at baseline (>< median FSS score at baseline), patients with high BL fatigue reported a significant and clinically important reduction of their severity of fatigue (FSS at BL: 49.7 ± 7.0 vs. FSS at FU: 32.0 ± 16.7; *P* *<* 0.01). In contrast, patients with low BL fatigue showed no further decrease in their FSS values. Finally, at follow-up, the severity of fatigue was comparable (*P* *=* 0.35) between patients with high versus low fatigue at baseline.

### 3.6 Comparison of changes in HRQoL and severity of fatigues in patients with versus without AIDS

Twenty HIV/HCV-coinfected patients (55.0%) had AIDS according to the CDC definition (Table 1, Supplementary Figure S1).^[38]^ When comparing baseline characteristics between patients with and without AIDS, no significant differences were found except for a significantly lower CD4+ nadir (median 77.5 [27.0–178.0] vs. 296.0 [213.0–734.0] cells/μL; *P* < 0.001) and a significantly lower current CD4+ count (median 315.0 [75.0–804.0] vs. 704.5 [231.0–985.0]; *P* *<* 0.01) in AIDS patients.

PCS was numerically—but not statistically significantly—higher in HIV/HCV-coinfected patients without AIDS. There seemed to be an improvement in physical health (PCS) with IFN-free therapy both in patients with and without AIDS, respectively. However, the improvements in physical health were only significant at FU (*P* *=* 0.01) in HIV/HCV-coinfected patients without AIDS and at mid-treatment (*P* *=* 0.03) in patients with AIDS.

Interestingly, mental health (mean MCS values at BL) was reportedly better among AIDS-patients (40.3 ± 6.0 vs. 35.6 ± 5.3; *P* *=* 0.04). While HIV/HCV-coinfected patients with AIDS did not report an amelioration of mental health (MCS), non-AIDS patients had a significant improvement in mental health (mean MCS values at BL: 35.7 ± 5.3 vs. FU: 40.7 ± 6.4; *P* *=* 0.04). Mental health in AIDS patients was similar to non-AIDS patients after eradication of HCV at follow-up (mean MCS at FU: AIDS: 40.3 ± 8.9 vs. non-AIDS 40.7 ± 6.4: *P* *=* 0.90).

As expected, AIDS patients reported a significantly higher severity of fatigue (mean FSS at BL: 42.5 ± 14.0 vs. 29.6 ± 10.0, *P* *<* 0.01). Patients with AIDS reported a significant stepwise reduction of severity of fatigue (FSS at BL: 42.5 ± 14.0; FSS at MID: 36.1 ± 14.8, *P* *=* 0.03; FSS at FU: 31.6 ± 15.7, *P* *=* 0.01).

### 3.7 Comparison of HRQoL and severity of fatigue between IFN- and RBV-free regimens and boceprevir-based triple therapy

**Figure 3:**
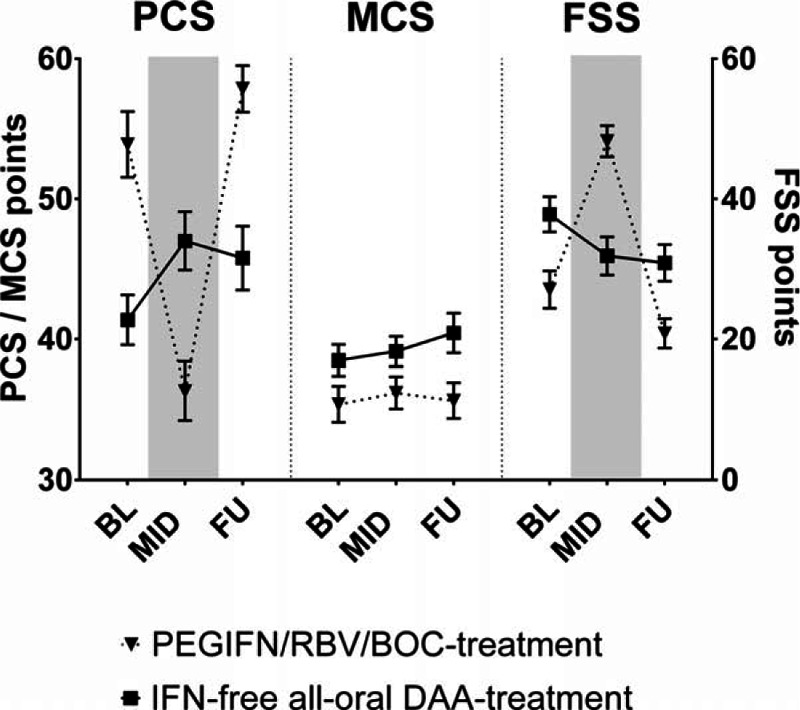
Comparison of physical and mental health, and fatigue at baseline, mid-treatment, and follow-up between HIV/HCV-coinfected patients treated with IFN-free direct acting antiviral versus Boceprevir/PEGIFN/RBV triple therapy; continuous variables shown as mean ± SEM. BL = baseline, BOC = boceprevir, DAA = direct acting antiviral, FSS = fatigue severity scale, FU = follow-up, MCS = mental component score, MID = mid-treatment, PCS = physical component score.

In comparison to the cohort of HIV/HCV-coinfected patients receiving (Table 2, Fig. 3, Supplementary Table S1) IFN-free regimens, patients in the BOC-based triple therapy group (13 men, 4 women) were younger (37.2 ± 8.8 vs. 49.9 ± 7.6 years; *P* < 0.001), had less advanced liver disease (17.6% vs. 93.9% with F3/F4), and showed a better BL physical health (mean PCS values at BL: 53.9 ± 9.7 vs. 41.4 ± 9.7; *P* < 0.001) and less fatigue (FSS at BL: 26.8 ± 10.8 vs. 37.8 ± 14.0; *P* *<* 0.01) as compared to the IFN-free study population.

**Table 2:**
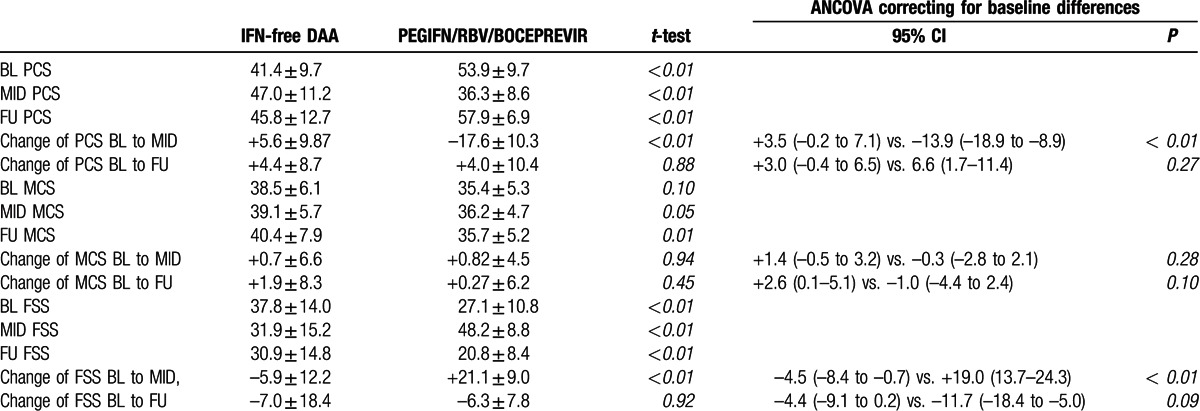
Comparison of HRQoL and fatigue during and after IFN-free DAA versus historical triple therapy.

There was a clear deterioration of physical health (mean PCS) and an increase in severity of fatigue (mean FSS) in HIV/HCV-coinfected patients receiving BOC-based triple therapy. During therapy (MID), the lines indicating physical health and severity of fatigue in patients treated with BOC-based triple therapy even crossed the PCS and FSS-curves of the “sicker” group of HIV/HCV-coinfected patients receiving IFN-free regimens. The physical health (PCS) and fatigue (FSS) at MID were significantly worse in the triple therapy group compared to the IFN-free group (MID PCS: 36.3 ± 8.6 vs. 47.0 ± 11.2, *P* *<* 0.01; MID FSS: 48.2 ± 8.8 vs. 31.9 ± 15.2, *P* < 0.001). This might be attributed to the pronounced deterioration of PCS and FSS during antiviral therapy with the BOC-based triple therapy (PCS: +5.6 ± 9.9 vs.: –17.6 ± 10.3, *P* < 0.001; FSS: –5.9 ± 12.2 vs. +21.1 ± 9.0; *P* < 0.001 at mid-treatment).

However, at FU, when all patients of the IFN-free group and 70.6% of the BOC/PEGIFN/RBV patients cleared the HCV infection—overall changes from BL to FU were comparable between both groups even after correcting for differences in BL values (PCS: +6.6 [95% confidence interval (CI): 1.7–11.4] vs. +3.0 [95%CI: –0.4 to 6.5], *P =* 0.27; FSS: –11.7 [95%CI: –18.4 to –5.0] vs. –4.4 [95%CI: –9.1 to 0.2], *P* *=* 0.09). However, physical health and severity of fatigue were still significantly better in the BOC-based triple therapy group (PCS: 57.9 ± 6.9 vs. 45.8 ± 12.7, *P* < 0.001; FSS: 20.8 ± 8.4 vs. 30.9 ± 14.8, *P* *<* 0.01), when compared to the IFN-free group.

Interestingly, while mean MCS-values were comparable at BL (IFN-free: 38.5 ± 6.1 vs. BOC: 35.4 ± 5.3, *P =* 0.10), patients receiving IFN-free treatment had significantly higher mean MCS values at MID (39.1 ± 5.7 vs. 36.2 ± 4.7, *P* *=* 0.05) and at follow-up (40.4 ± 7.9 vs. 35.7 ± 5.2, *P* *=* 0.01) when compared to triple therapy patients, indicating improvements in mental health in the IFN-free group following treatment initiation.

## 4 Discussion

Novel IFN- and RBV-free regimens are extremely effective and safe and thus, allow treatment of HIV/HCV-coinfected patients with advanced liver disease and severe comorbidities.^[16,39]^ Many of these conditions were contraindications to traditional IFN-based therapy, resulting in a shift toward treatment of sicker patients with novel regimens.^[21]^ According to current EASL guidelines,^[7]^ HCV treatment is indicated in all patients with chronic HCV infection and should be prioritized in patients with advanced liver disease (as defined as liver fibrosis grade ≥F3), HIV coinfection, patients with debilitating fatigue as well as in individuals with high risk of transmission. According to these guidelines, the HIV/HCV-coinfected patients included in this study had several indications for prioritized access to treatment.

Among patients with or at risk for CSPH, 4 patients had portosystemic collaterals, whereas elevated blood ammonia levels were only observed in one patient. Overt HE has strong implications on HRQoL.^[40]^ Only one patient had overt HE at BL. Therefore, overt HE did not significantly affect the results of our study. However, we did not perform neuropsychological testing. Thus, covert HE might have contributed to HRQoL impairments.^[40]^

Similar to previous reports,^[12,41]^ our cohort of HIV/HCV-coinfected patients also showed significant impairments of physical and mental health as well as pathological fatigue values, when compared to the general population.

Recent studies in HCV-monoinfection^[42]^ and HIV/HCV coinfection^[41]^ showed that patients—even though there was a moderate decrease of HRQoL during treatment with SOF/RBV—reported an improvement in HRQoL scores after achievement of SVR. Those studies are not directly comparable to our study, since RBV-containing regimens cause anemia, which impairs HRQoL.^[12,43]^ In contrast to IFN/RBV-containing regimens,^[12]^ the avoidance of RBV—as in this study—elucidates improvements of physical health and decreases of fatigue occurring already during therapy. Our group of HIV patients also reported significant improvements in HRQoL at FU, that is, after eradication of HCV coinfection by IFN- and RBV-free regimens. This finding is consistent with the results of a recently published study by Younossi et al.^[43]^ reporting HRQoL changes in a very large cohort of HCV-monoinfected patients with compensated liver disease treated with second-generation DAAs with or without RBV. This study also found that HRQoL improvements occurred very early in the treatment period.^[43]^ Previous studies failed to demonstrate a clear correlation between the deterioration in HRQoL and hepatic necroinflammatory activity.^[44,45]^ Thus, the exact mechanism by which the eradication of HCV infection—that is, the cessation of hepatic necroinflammatory activity—leads to improvements of HRQoL, remains to be established. Another potential explanation for the early improvements in HRQoL—when HCV viremia is cleared—might be a potential neuropathogenic role of HCV. However, while this has been discussed for years,^[46,47]^ there is still no convincing evidence for a neurotropic effect of HCV infection.

In our study, almost half of patients (45.5%) showed clinically important improvements of PCS from BL to FU, but only one third of patients (33.3%) reported an improvement in MCS and almost half of patients (45.5%) did not report a clinically important change in MCS over the whole study period, indicating that other factors such as social status,^[48]^ work satisfaction and believe may play a more important role for mental health in HIV patients. Nevertheless, patients reported substantial improvements in HRQoL during and after IFN-free therapy—especially in the subscales bodily pain (BP) and role emotional (RE). However, we also noted a decrease in the SF-36 subcategory mental health (MH), which is consistent with data presented at the AASLD Liver Meeting 2015.^[49]^ In contrast, overall mental health—as reflected by the MCS (including all SF36-subscores with a higher impact of vitality, social functioning, role emotional, and mental health)—tended to increase in HIV/HCV-coinfected patients without AIDS. However, we do not have a universal explanation for the discrepancy between the decrease in the mental health subcategory and the trend toward increasing MCS scores in the patient-reported SF-36 questionnaire.

In our study, HIV/HCV-coinfected patients with significant fatigue at BL showed more pronounced FSS improvements. This is in line with a previous study in HCV monoinfection^[50]^ and demonstrates, that this finding also applies to HIV-positive patients. Importantly, our results support the EASL recommendation to prioritize HCV treatment in patients with debilitating fatigue.^[7]^

However, this study is the first that directly compares changes in HRQoL between patients treated with first-generation DAA-based triple therapy and IFN- and RBV-free second-generation DAA regimens. In the meantime, it has become apparent that treatment with BOC-based triple therapy leads to even more pronounced side-effects compared to PEGIFN/RBV alone.^[51]^ Accordingly, our patients treated with a BOC-containing regimen—even though being much younger and “healthier” at BL—reported worse HRQoL at mid-treatment than the “sicker” IFN-free treatment group. Interestingly, even after correcting for baseline differences by using the ANCOVA method, we found that both patient groups achieved comparable PCS, MCS, and FSS changes from the respective BL to FU values. As previously suggested,^[43]^ this indicates that even patients with advanced liver disease have the potential for significant improvements of HRQoL with novel HCV regimens therapy.

The main limitations of our study are its retrospective design and the limited number of patients. Moreover, although we used ANCOVA to account for differences at BL, we cannot exclude that the severity of liver disease at BL affects the dynamics of HRQoL and fatigue during treatment.

In conclusion, IFN- and RBV-free therapy leads to substantial improvements in HRQoL and fatigue levels in HIV/HCV-coinfected patients with advanced liver disease. Already during therapy, IFN- and RBV-free treatment increases physical health and decreases severity of fatigue. HIV/HCV-coinfected patients with significant fatigue levels at BL show most pronounced decreases in fatigue severity. Thus, they should be prioritized for HCV treatment. Importantly, HCV eradication leads to sustained and clinically relevant improvements in physical health in 45% and in mental health in 33% of HIV/HCV-coinfected patients with advanced liver disease.

## Supplementary Material

Supplemental Digital Content
