# Supplementary material for: Interferon-free regimens improve health-related quality of life and fatigue in HIV/HCV-coinfected patients with advanced liver disease: A retrospective study
Source: Medicine (Baltimore). 2016 Jul 8;95(27):e4061. doi: 10.1097/MD.0000000000004061 (PMC5058819; doi:10.1097/MD.0000000000004061)
Supplement: Supplemental Digital Content [file medi-95-e4061-s001.doc]

Supplementary Table-S1. Baseline characteristics of the historic control (HIV/HCV-coinfected patients treated with PEGIFN/RBV in combination with BOC)

| **Sex, male/female (% male)** | 13/4 (76.5%) |
| --- | --- |
| **Age at therapy (years ± SD)** | 37.2 ± 8.8 |
| **HCV-Genotype** |  |
| ***Genotype 1, n*** | 17 |
| **Prior / actual alcohol abuse, n (% with abuse)** | 8 (47.1%) |
| **cART, n (% receiving cART)** | 17 (100.0%) |
| **Advanced fibrosis, n F3/F4 (% F3/F4)** | 3 (17.6%) |
| **High HCV-RNA, n (% with HCV-RNA >6*10^6 copies/µL)** | 15 (88.2%) |
| **CD4-Nadir (cells/U) median (Min-Max)** | 174.0 (37.0-443.0) |
| **Hemoglobin (g/dl), mean ± SD** | 14.4 ± 2.0 |
| **AST (U/l), median (Min-Max)** | 50.0 (15.0-260.0) |
| **ALT (U/l), median (Min-Max)** | 66.0 (24.0-291.0) |
| **CD4 cell count (cells/U), median (Min-Max)** | 486.0 (267.0-740.0) |
| **PCS, mean ± SD** | 53.9 ± 9.7 |
| **MCS, mean ± SD** | 35.4 ± 5.3 |
| **Severity of fatigue, mean ± SD** | 26.8 ± 10.8 |

**Supplementary Figure-S1:** Subgroup analyses of physical and mental health, and severity of fatigue at baseline, mid-treatment and follow-up in patients with and without AIDS; continuous variables shown as mean ± SEM; Abbreviations: PCS (physical component score), MCS (mental component score), FSS (fatigue severity scale), BL (baseline), MID (mid-treatment), FU (follow-up)


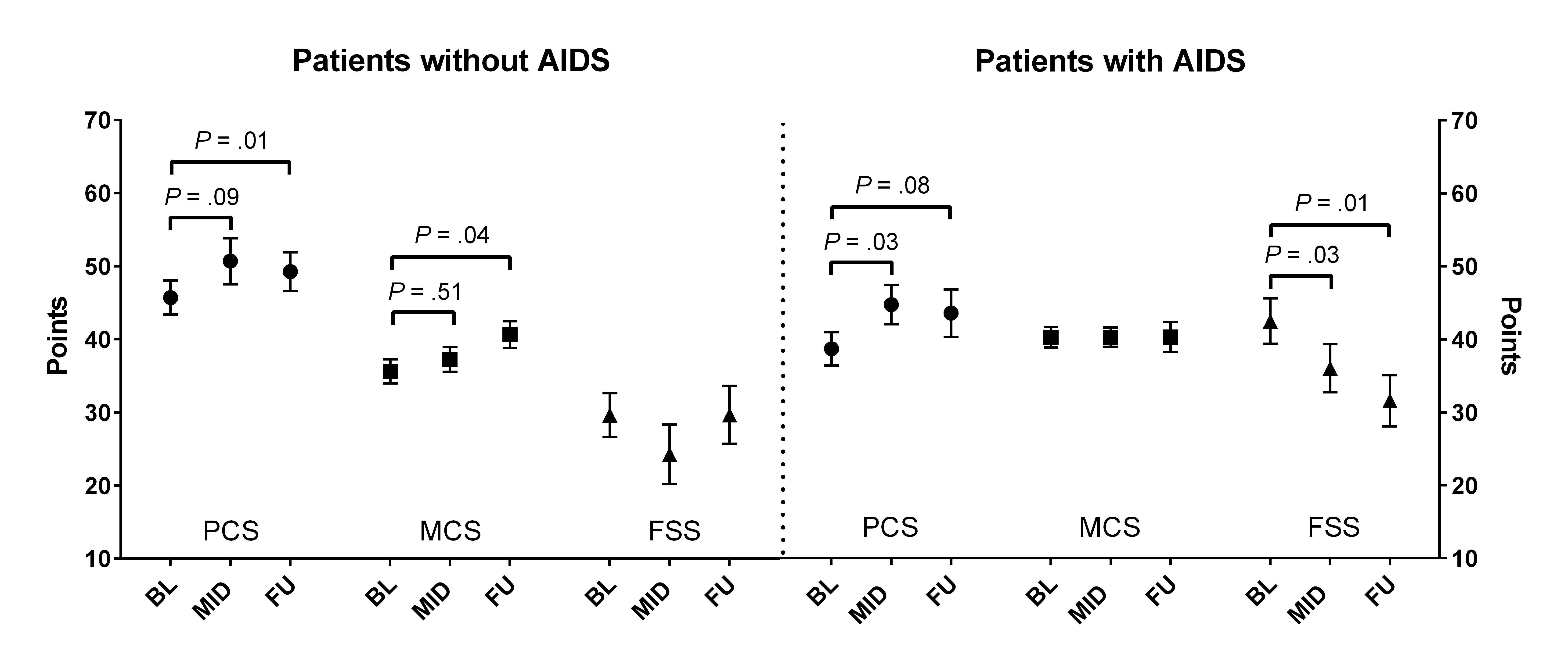
s
